# Patterns and treatment outcomes of primary bone tumors in children treated at tertiary referral hospital, Ethiopia

**DOI:** 10.1186/s12885-024-12169-x

**Published:** 2024-03-28

**Authors:** Temesgen Lingerih, Sewagegn Yeshiwas, Abdulkadir Mohamedsaid, Gashaw Arega

**Affiliations:** 1 Department of Pediatrics and Child Health, Debretabor University, School of Medicine, Debretabor, Ethiopia; 2 Department of Pediatrics and Child Health, Addis Ababa University, School of Medicine, Addis Ababa, Ethiopia

**Keywords:** Primary bone tumors, Overall survival, Event-free survival, Kaplan Meier

## Abstract

**Background:**

Bone tumors account for approximately 6% of all cancers in children. Malignant bone tumors, commonly occurring in children and adolescents, are associated with high mortality and morbidity. The overall survival of children with primary malignant bone tumors is affected by the stage of disease, time of diagnosis, and treatment response. Despite advanced treatment modalities with chemotherapy, surgery, and radiotherapy, bone tumor is the third leading cause of death in children with malignancy. Patients with metastatic disease at diagnosis have poor outcomes compared to localized disease at presentation. The 5-year Overall Survival and event-free survival in children with primary malignant bone tumors were 85.2% and 69.2%. The study aimed to assess the clinicopathological profile and treatment outcomes of children with primary malignant bone tumors in our setup.

**Materials and methods:**

A hospital-based cross-sectional study was conducted on 95 children who met the inclusion criteria through structured questionnaire. The collected data were analyzed using a statistical package for social sciences (SPSS) version 25. *P*-value < 0.05 was considered to be statistically significant. Kaplan Meier survival estimate was used for overall and event-free survival analysis.

**Results:**

A total of ninety-five patients met the study inclusion criteria and the median age at diagnosis with primary malignant bone tumors was 10 years, with an interquartile range of 8–12 years. The duration of the illness from the onset of symptoms to the oncologic treatment center ranges from three weeks to 2 years with a mean duration of five months. Swelling was the commonest presenting symptom accounting for 95.8% (*n* = 91). Lower extremity was the commonest primary site of involvement accounting for 55.8% (*n* = 53) of children with primary malignant bone tumors. Osteosarcoma was the commonest malignant bone tumor constituted 66.3% (*n* = 63), followed by Ewing sarcoma at 33.7% (*n* = 32).

About 41.2% (*n* = 39) of children had metastatic disease at presentation and the lung was the commonest site of distant metastasis. The Kaplan Meier survival estimate analysis showed the 1-year and 5-year overall survival probabilities for all pediatric primary malignant bone tumor patients were 65% (95% CI: 0.3–0.56) and 38% (95% CI:0.19–0.47) respectively. The 1-year and 5-year event-free survival probabilities were 55% (95% CI: 0.32–0.73) and 33% (95% CI: 0.10–0.59). The stage of the disease at presentation had a significant association with the outcome (*p* = 0.023).

**Conclusion:**

Our study showed the mean duration of the illness from the onset of symptoms to the oncologic treatment center was 5 months ranging from 3 weeks to 2 years. More than one-third of the presented with metastatic disease at presentation. The 1-year and 5-year overall survival (OS) probabilities of children with primary malignant bone tumors were low in our setup compared to other studies.

## Introduction

Bone tumors arise from bone and closely related structures in the skeletal system [1, 2]. Primary malignant bone tumors are more common in children and young adolescents than in the adult population, constitute approximately 6% of all cancers in children, and are highly associated with high mortality and morbidity [3–5]. Osteosarcoma is the most common malignant bone tumor in childhood followed by Ewing sarcoma, boys are slightly more affected than girls (1.5: 1) and the peak incidence is in the second decade of life [6–8]. A study done in Germany from 1987–2011 showed the median age of occurrence of primary bone tumors was 10–14 years old with an average age of 12 years. The incidence rates of specific bone sarcomas are age-related; the first well-defined peak occurs during the second decade of life. Up to 56% of malignant bone tumors will arise around the knee in the distal femur and tibial bone in those under the age of 20 years. The other most common site is the pelvis, which is numerically the most common site of presentation for both Ewing sarcoma and chondrosarcoma [4, 9].

About 80% of osteosarcomas occur in the extremities; the distal femur, proximal tibia, and proximal humerus are the commonest sites of occurrence. Whereas, Ewing sarcoma family tumors are evenly distributed between the axial and appendicular skeleton [7, 10–12]. In a review of the histopathological pattern of primary bone tumors and tumor-like lesions in Ile-Ife, Nigeria; Osteosarcoma was the most common primary malignant bone tumor accounting for 42%. About 42.86% occurred in the distal third of the femur, 14.28% in the distal tibia, 14.28% in the proximal tibia, 9.52% in the mandible, and the proximal third of the femur, the maxilla, humerus, and calcaneus 4.76% each.

In a pathology review of 335 patients with bone tumors in Ethiopia; 158 (47%) had benign tumors and 177 (53%) had malignant tumors. Osteochondroma was the most common benign bone tumor and osteosarcoma constituted 62% of all primary malignant bone neoplasms, followed by Ewing sarcoma, and chondrosarcoma. The most common age groups affected by primary bone tumors were 10–29 years and the most common locations of presentation for primary malignant bone tumors were the distal femur and proximal tibia [13, 14].

Genetic factors; such as Li-Fraumeni syndrome, RB gene mutation, environmental factors, and previous radiation therapy may increase the risks of developing primary bone tumors [15].

Bone tumors in children were the third leading death of malignancy and the trend of survival rate was increasing with an overall survival rate of 79% with a significant impact on the quality of life with high mortality and morbidity [16, 17]. The mean survival times for patients with osteosarcoma, and Ewing sarcoma were approximately 54.9 and 58.1 months respectively [18–20]. Despite the advanced combined treatment modalities using chemotherapy, surgery, and radiotherapy, malignant bone tumors affect the quality of life of children and contribute significantly to childhood cancer morbidity and mortality. The five-year survival rate and event-free survival for localized malignant bone tumors were 81% and 68% respectively, whereas the overall survival for metastatic bone tumors was 42% [21–24].

Based on Estimates of Cancer Incidence in Ethiopia in 2015 [25], bone and cartilage tumors accounted for 7% of all childhood cancers. However, there was no well-documented data about primary bone tumor patterns and survivals in our setup. Thus, this study aimed to provide an overview of the histopathological pattern of primary bone tumors, describing the anatomic sites, and assessing the outcomes of the primary bone tumors treated at Tikur Anbessa Specialized Hospital, Addis Ababa, Ethiopia.

## Methodology

### Study setting

The study was conducted at the Department of Pediatrics and Child Health, Haemato-Oncology Unit, Tikur Anbessa Specialized Hospital, Addis Ababa, Ethiopia. Tikur Anbessa Specialized Hospital is the largest tertiary hospital in the country established in 1974, and administered by Addis Ababa University. Tikur Anbessa Specialized Hospital is one of the largest pediatric haemato-oncology treatment centers in the county, and a separate unit for children with cancer began in March 2013 with initiatives taken by the International Network for Cancer Treatment and Research, USA (INCTR-USA) in collaboration with George Town University Hospital. The pediatric haemato-oncology wards have 42 inpatient beds dedicated to pediatric cancer patients and the unit gives both inpatient and outpatient services for more than 800 patients every month.

### Sampling

All radiologically and pathologically confirmed primary bone tumors in children under the age of 15 years from January 1, 2014, to June 30, 2022, were included. Children with secondary metastatic bone tumors, tissue biopsies revealing benign bone tumors, and patients with incomplete records were excluded. A total of ninety-five (*n* = 95) patients met the inclusion criteria and were included in our study. A hospital-based cross-sectional study was carried out through a self-administered structured questionnaire. Data were collected from July 1, 2022, to September 30, 2022.

### Data collection and data analysis

Data were collected by the principal investigator and trained General Practitioners using structured self-administered questionnaires. The study questionnaires had four parts: Part I was about the socio-demographic characteristics of the study participants, Part II was about the clinical profile of patients at presentation, Part III was about the diagnosis and diagnostic investigations, and Part IV was about the treatment profiles and outcomes of primary malignant bone tumors in children treated at Tikur Anbessa Specialized Hospital.

After selecting the study cases, the data was collected from the registration log book, the patient card, and the follow-up chart by the data collectors. The administered questionnaire encompasses the socio-demography profile, clinical profile, and outcome. ODK version 2022.3.3 software was used to collect the data along with the Kobo Toolbox server to store the collected data. Data was entered into Epi data version 3.1 and exported to SPSS version 25 for analysis. *P*-value < 0.05 was considered to be statistically significant. The Kaplan Meier survival was used to estimate the first, and fifth year overall and event-free survival analysis.

Overall survival (OS) represents the time from the date of first diagnosis to the date of last follow-up or death from any cause.

Event-free survival is the time duration after treatment completion for a primary bone tumor that the patient remains free of any events.

Events include treatment abandonment, relapse, and death.

### Data quality control and management

To ensure the quality of data, the structured questionnaire checklists were tested on 5% of the sample. Problems highlighted during the pre-test were corrected before the start of the data collection. Each question was properly coded; continuous cross-checking was done both during the pre-test and data collection period by the principal investigator. The collected data were checked for completeness and consistency on each day of data collection.

### Ethical approval

Ethical approval was obtained from the Research and Publication Committee of Pediatrics and Child Health Department (DRCP), School of Medicine, College of Health Sciences, Addis Ababa University. Confidentiality was fully maintained during data collection and analysis. Participants would be anonymous during the dissemination of results.

## Results

### Socio-demographic characteristics of patients

During the study period, a total of ninety-five patients met the study inclusion criteria and were included in the study. Our study showed that 52.6% (*n* = 50) were males and 47.4% (*n* = 45) were females with male to female ratio of 1.1: 1. The median age at diagnosis was 10 years, with an interquartile range of 8–12 years. Nearly half of the children, 48.4% (*n* = 46) were between the ages of 5 and 10 years and 45.2% (*n* = 43) were between 11- 15 years. Most of the children; 87.4% (*n* = 83) came from out of Addis Ababa, the capital city of the country (Table 1).

**Table 1:** socio-demographic characteristics of pediatric primary malignant bone tumor patients (*n* = 95)

Variable	**Frequency**	**Percent (%)**
Age category		
< 5 years	5	5.3
5–10 years	46	48.4
11–15 years	43	45.2
≥ 15 years	1	1.1
Sex		
Male	50	52.6
Female	45	47.4
Residence		
Oromia	29	30.5
Addis Ababa	12	12.6
SNNPR	21	22.1
Amhara	24	25.3
Other	9	9.5

*SNNPR* Southern Nations Nationalities and Peoples' Region

### Clinical profile of children with primary bone tumors at Tikur Anbessa Specialized Hospital

The duration of the illness from the onset of symptoms to the oncologic treatment center ranges from three weeks to 2 years with a mean duration of 5 months. The average duration of a delay from the primary referring institute to the oncologic treatment center was 5.8 ± 8.2 SD days and the average time of treatment initiation after visiting the treatment center was more than 1 month (35.7 days).

**Fig. 1:**
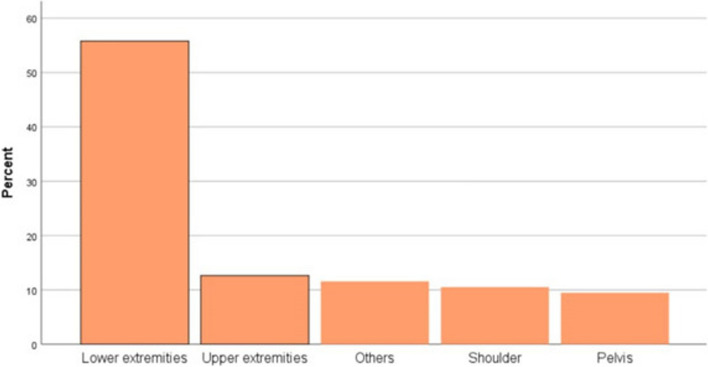
Primary site of occurrence in children with primary malignant bone tumors

Swelling was the commonest presenting symptom accounting for 95.8% (*n* = 91). The commonest primary site of involvement was the lower extremity accounting for 55.8% (Fig. 1). Anthropometric nutritional assessment showed that 14.7% (*n* = 14) were severely acutely malnourished and 25.3% (*n* = 24) were moderately malnourished at presentation (Table 2).

**Table 2:** Clinical characteristics of children’s primary malignant bone tumor patients (*n* = 95)

Variable	**Frequency**	**Percent (%)**
Duration of symptoms in days (median + IQR)	155 ± 165	
Presenting symptoms		
Swelling	91	95.8
Pain	51	56.7
Constitutional symptoms	21	22.1
Fractures	1	1.1
Others (limping, wound, falling)	8	8.4
Site of lesion Lower extremity	53	55.8
Upper extremity	12	12.6
Shoulder	10	10.5
Pelvis	9	9.5
Other site (chest wall, neck, face, spine)	11	11.6

*IQR* Interquartile range

### Diagnosis and diagnostic work-up

**Fig. 2:**
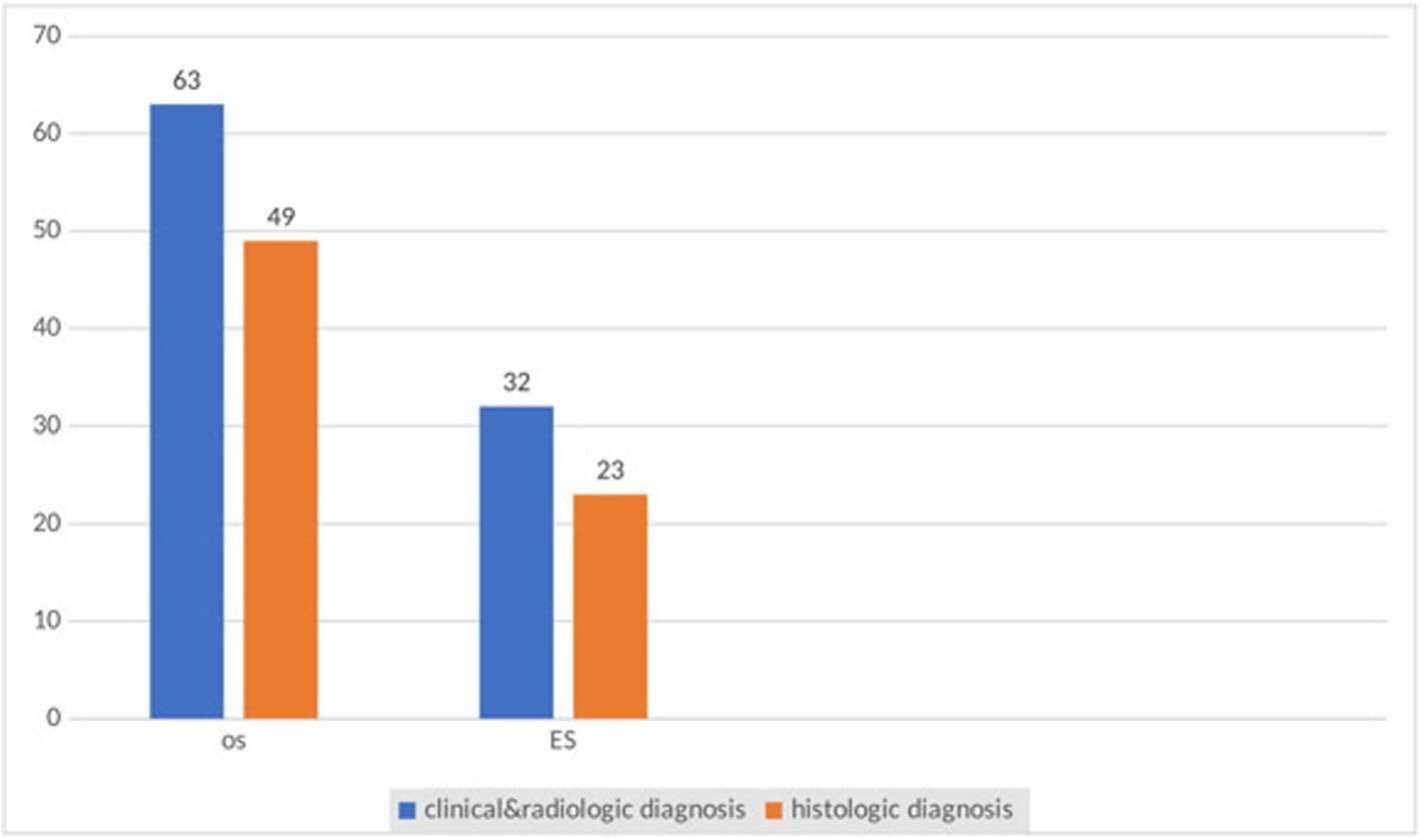
Diagnosis of primary malignant bone tumors in the oncologic treatment center. OS: Osteosarcoma; ES: Ewing sarcoma

**Fig. 3:**
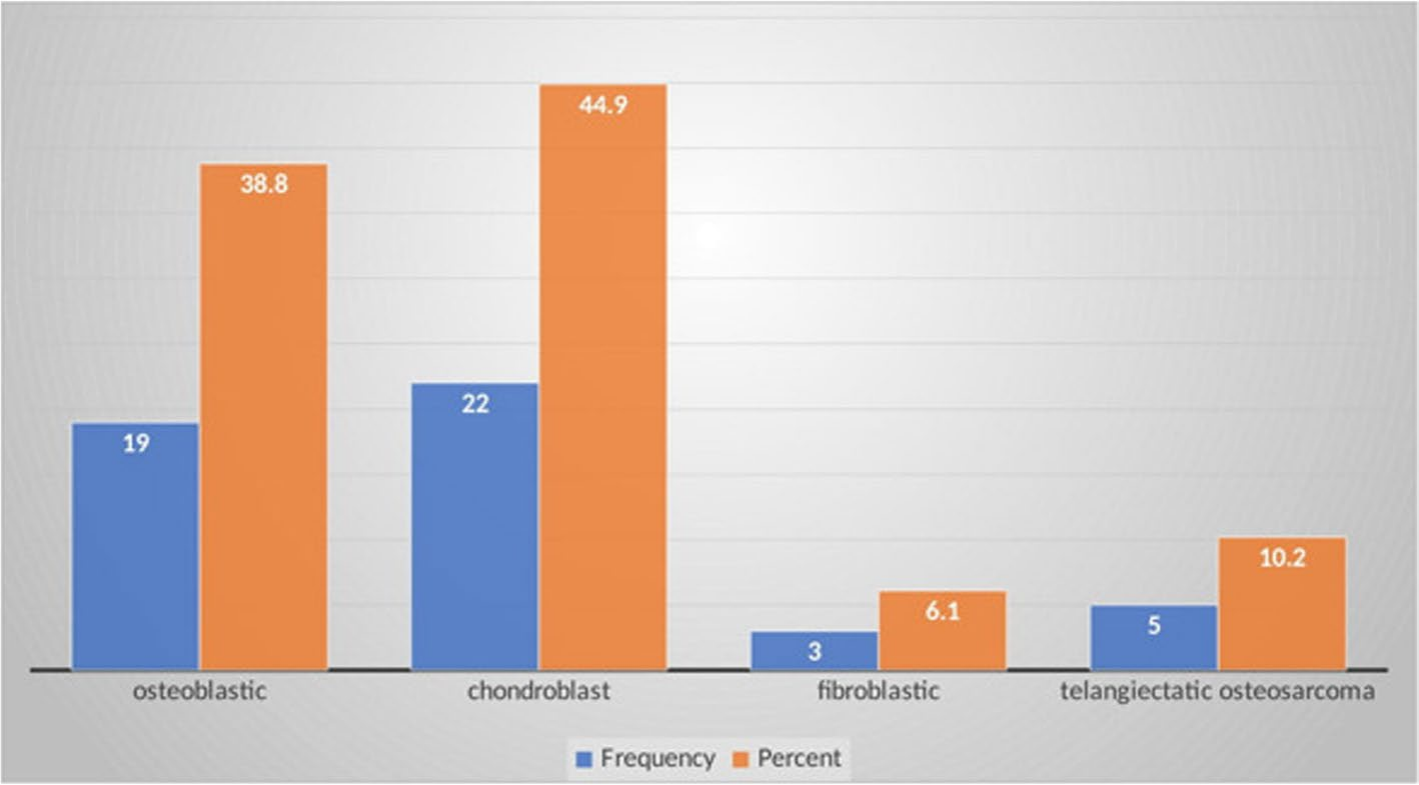
Histologic subtypes of Osteosarcoma in children with primary malignant tumor in Tikur Anbessa Specialized Hospital

Our study showed, that 58.1% (*n* = 54) of the patients had localized disease, and 41.2% (*n* = 39) of the patients presented with metastatic disease at presentation commonly to the lungs. More than one-third of patients; 40.5% (*n* = 32) had chest metastases on the Chest CT scan, 17.9% (*n* = 10) had chest metastases on Chest X-ray, 29.2% (*n* = 7) had abdominal-pelvic metastases and 21.7% (*n* = 5) of Ewing sarcoma had bone marrow involvement. Sixty-three (66.3%) patients were diagnosed with Osteosarcoma and 32 (33.6%) patients were diagnosed with Ewing sarcoma (Table 3). Only 75.8% (*n* = 72) had a histologic diagnosis of bone tumors. Histologic diagnosis was confirmed for 68% (*n* = 49) and 32% (*n* = 23) of osteosarcoma and Ewing sarcoma respectively (Fig. 2). The commonest histologic sub-type of Osteosarcoma in our study was chondroblastic osteosarcoma accounting for 44.9% (*n* = 22) of patients (Fig. 3).

**Table 3:** Diagnosis and diagnostic work-up of children with a primary malignant bone tumor

Variable	**Frequency**	**Percent (%)**
WBC (*10^9^/L) (median + IQR)	10	10 ± 9.3
Neutrophil (%)	60.4	60.4 ± 74.3
Platelets (*10^9^/L) (median + IQR)	410	410 ± 216
Radiologic investigation(*n* = 95)		
bone MRI	51	53.7
bone x-ray	38	40
bone CT-scan	33	34.7
Metastatic workup		
Yes	93	97.9
No	2	2.1
Types of metastatic workup(*n* = 93)		
Chest CT scan	79	83.2
Finding of metastasis	32	40.5
Chest X-ray	56	58.9
The finding of metastasis in X-ray	10	17.9
Abdominal Ultrasound	44	46.3
Finding of metastasis	3	6.8
Abdominal CT-scan	24	25.3
Finding metastasis	7	29.2
Bone marrow biopsy	23	71.8
Finding of metastasis	5	21.7
Localized Disease at Diagnosis	54	58.1
Metastatic Disease at Diagnosis	39	41.9
Osteosarcoma localized	37	58.7
Metastasis	24	38.1
Ewing sarcoma localized	17	53.1
Metastasis	15	46.9

*U/S* Ultrasound, *SD* Standard deviation, *IQR* Interquartile range, *WBC* White blood cell count

### Treatment profile of children with primary bone tumors

Sixty-eight of the patients (71.6%) started treatment; 64.7% (*n* = 44) with curative intent and 35.3% (*n* = 24) with palliative treatment intent. Chemotherapy was the most common type of treatment modality offered for 98.5% (*n* = 67) of patients, whereas surgical treatment was done for 30.5% (*n* = 29) and 9.4% (*n* = 3) of Ewing sarcoma patients received radiotherapy. Chemotherapy was administered as first-line treatment for 82.4% (*n* = 56) patients, whereas surgical treatment was offered as first-line therapy for 17.6% (*n* = 12). More than half of the patients, 62.7% (*n* = 42), received the Osteosarcoma chemotherapy protocol. Ewing sarcoma patients were treated with VCD/IE, VCD, and other (VAC, Metronomic) protocols; 22.4% (*n* = 15), 5.9% (*n* = 4), and 10.3% (*n* = 7) respectively. Nearly half of the patients, 32 (47.1%) completed the chemotherapy. Among the surgically treated patients, 79.3% (*n* = 23) were amputated, and debulking of the mass was done for two (6.9%) patients (Table 4).

**Table 4:** Treatment-related characteristics of pediatric malignant bone tumor patients

Variable	**Frequency**	**Percent (%)**
Treatment initiated		
No	27	28.4
Yes	68	71.6
Therapeutic intent (*n* = 68)		
Curative	44	64.7
Palliative	24	35.3
Treatment completed (*n* = 68)		
No	36	52.9
Yes	32	47.1
Reason for not completing treatment (*n* = 35)		
Death	5	14.3
Lost to follow up	18	51.4
Left against medical advice	9	25.7
Palliative care (due to progression)	3	8.6
Type of protocol (*n* = 67)		
OS1&OS2 protocol	42	61.8
VCD/IE protocol	15	22.1
VCD protocol	4	5.9
Other (VAC, PO palliative)	7	10.3

*OS1* Cisplatin and doxorubicin, *OS2* Cyclophosphamide and Etoposide, *VCD/IE* Vincristine cyclophosphamide doxorubicin ifosfamide and etoposide, *VAC* Vincristine Actinomycin and cyclophosphamide

### Treatment outcomes of children with primary bone tumors

**Fig. 4:**
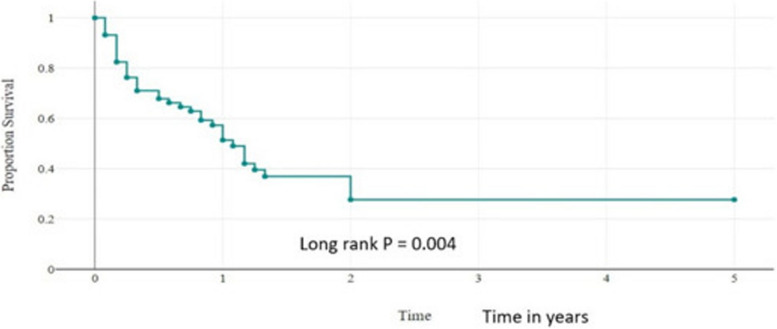
Overall survival outcomes pediatrics primary malignant bone tumors in years

**Fig. 5:**
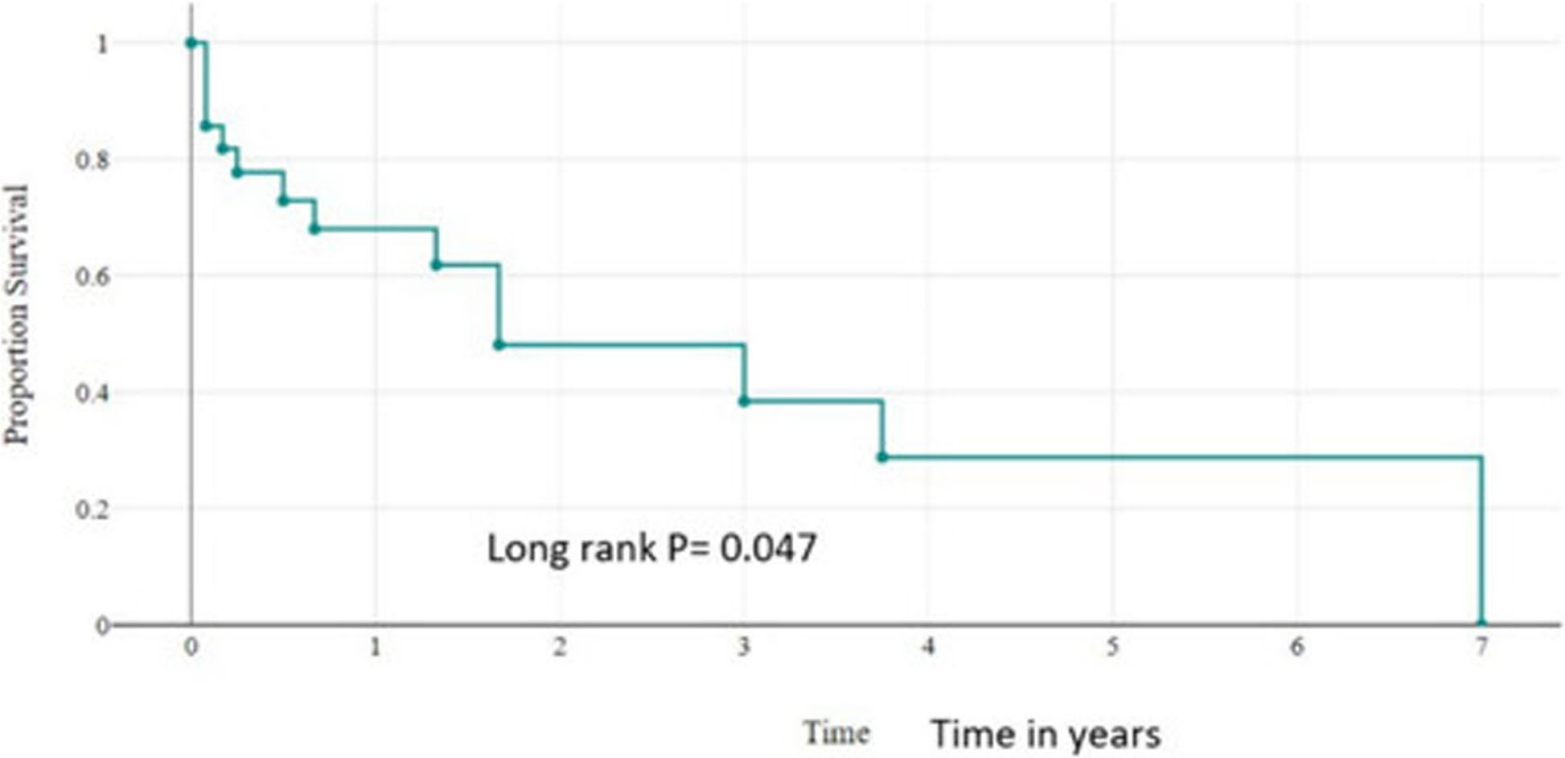
Event-free survival (EFS) of children with primary malignant bone tumors in years

During the study period, the survival status showed that 46.3% (*n*=44) were died, 27.4% (*n*=26) were alive and for 26.3% (*n*=25) the status was unknown. After completion of chemotherapy, 46.9% (*n*=15) had no new event, 43.8% (*n*=14) had relapsed, and for 9.3% (*n*=3) the status was unknown, lost to follow-up. Kaplan Meier analysis showed that the median overall survival time for all pediatric patients who had primary malignant bone tumors was around 1 year (95%CI: 0.8-3.12). The 1-year and 5-year overall survival probabilities for all pediatric primary malignant bone tumor patients were 65% (95%CI:0.3-0.56) and 38% (95%CI:0.19-0.47) respectively (Fig. 4). The 1-year and 5-year event-free survival probabilities were 55 % (95 CI: 0.32-0.73) and 33% (95% CI: 0.10-0.59) (Fig. 5).

### Factors affecting treatment outcomes

To identify the associated factors with survival status, a chi-square test of independence was performed. Statistical significance was considered at a level of significance of 5%. Our finding revealed that survival status was independent of age, sex, duration of illness, and primary site of occurrence (*p* = 0.6, 0.78, 0.39, and 0.07) respectively, whereas it was dependent on the stage of the disease at diagnosis (*p* = 0.023), and the histology of the tumor; either osteosarcoma or Ewing sarcoma (*P* = 0.05) (Table 5).

**Table 5:** Factors affecting treatment outcomes in children with primary malignant bone tumors

		**Survival status**			***P*-value**
		**Died**	**Alive**	**Unknown**	
**Age**	Under 5 years	3(60%)	1(20%)	1(20%)	0.687
	5 to 10 years	22(47.8%)	10(21.7%)	14(30.4%)	
	11 to 15 years	19(43.2%)	15(34.1%)	10(22.7%)	
**Sex**	Male	23(46%)	15(30%)	12(24%)	0.784
	Female	21(46.7%)	11(24.4%)	13(28.9%)	
**Duration of illness**	< 1 year	40(44.9%)	24(27%)	25(28.1%)	0.392
	1 to 5 years	2(50%)	2(50%)	0(0%)	
**Primary site occurrence**	lower extremities	23(43.4%)	17(32.1%)	13(24.5%)	0.078
	pelvis	2(22.2%)	2(22.2%)	5(55.6%)	
	shoulder	8(80%)	1(10%)	1(10%)	
	upper extremities	8(66.7%)	1(8.3%)	3(25%)	
	others	3(27.3%)	5(45.5%)	3(27.3%)	
**Histologic finding**	osteosarcoma	25(39.7%)	22(34.9%)	16(25.4%)	0.05
	Ewing sarcoma	19(59.4%)	4(12.5%)	9(28.1%)	
**Stage of disease at diagnosis**	localized	21(37.5%)	21(37.5%)	14(25%)	0.023
	metastasis	23(59%)	5(12.8%)	11(28.2%)	

**Fig. 6:**
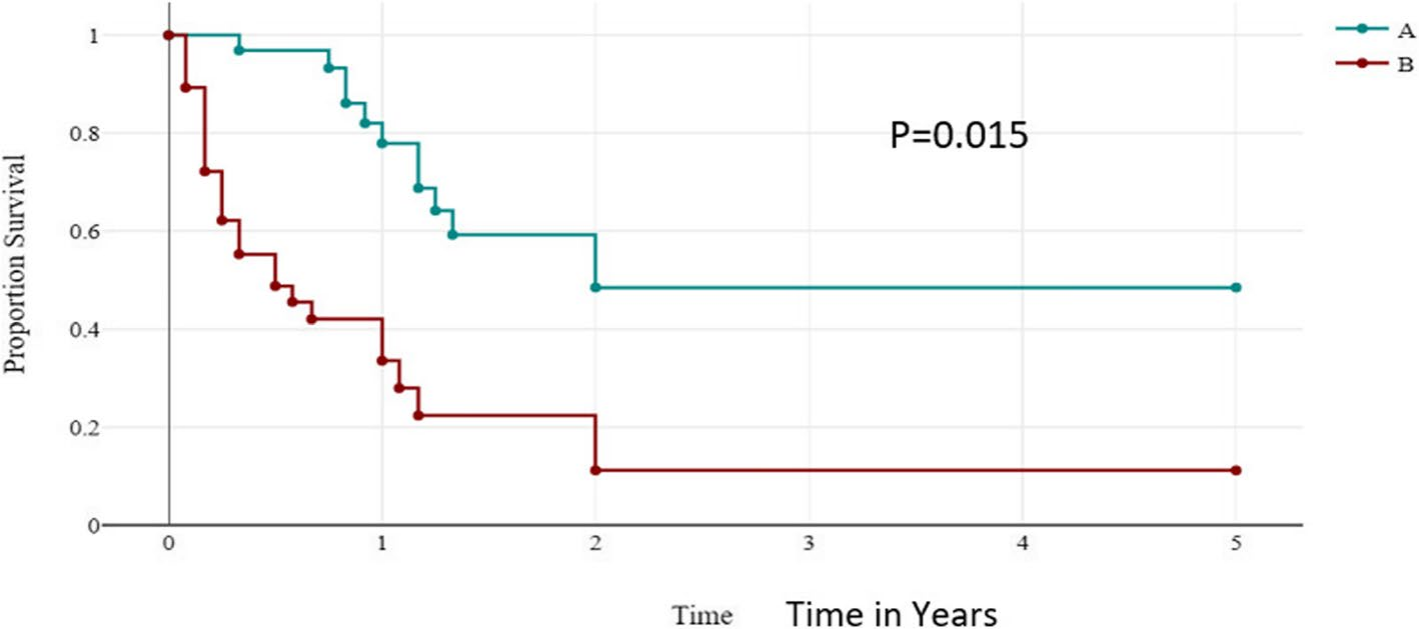
Survival rate distribution of Group A and Group B time in years. Group A = Chemotherapy completed; Group B = Chemotherapy not completed/started

A log-rank test was calculated to see if there was a difference between those treatments completed and those treatments not completed or started in terms of the distribution of time to survival and death occurrence and showed that there is a significant difference in groups in terms of the distribution of time survival (*P* ≤ 0.015). Those children who completed the chemotherapy had better survival than those who didn’t complete or started at all (Fig. 6).

## Discussion

Primary malignant bone tumors are one of the common malignant solid tumors in childhood and adolescence age, but the clinical profile and outcome status were not well studied in Low and Middle-Income Countries (LMICs). Most studies revealed that the median age of diagnosis was 10–14 years, with males slightly more affected than females with a 1.5:1 ratio [9, 24, 26].

Our study showed that the median age of diagnosis of primary malignant bone tumor was 10 years with an interquartile range of 8–12 years, which showed slight occurrence at an earlier age compared to another study done in Germany, which showed a median age of 10–14 years [9]. The difference might be due to the participants in our study being under 15 years old.

The duration of illness from the onset of symptoms to the oncologic treatment center ranges from 3 weeks to years with a mean duration of 5 months. In our study, the major clinical presentation was swelling (95.8%), followed by pain (53.7%), whereas pain was the most common presentation in a study done in India [2]. The site of occurrence is similar to other studies but variation of clinical presentation may be due to age differences and benign lesions were also included in the other studies [2, 26–28].

In our study, 41.9% (*n* = 39) were diagnosed with advanced (Metastatic) disease, and 58.1% (*n* = 54) with localized disease at presentation. An institution-based retrospective study done at St. Jude Children’s Research Hospital Studies showed 71% of bone tumor patients had localized disease and 29% had metastasis at the diagnosis. The high incidence of metastatic presentation at diagnosis in our setup might be due to a lack of awareness of cancer, delayed seeking of medical care (delayed presentation), delayed referral, and delayed diagnosis.

Osteosarcoma was the commonest primary malignant bone tumor accounting for 66.3% (*n* = 63) followed by Ewing sarcoma 33.7%) in our study, similar results were found in the study done in Ethiopia; Osteosarcoma accounts for 62%, followed by Ewing sarcoma; 15.2% [13] and in South Iran, which was done among 100 patients; 57 patients were diagnosed with Osteosarcoma and 43 patients with Ewing sarcoma [29].

The lower extremity was the most common primary site of involvement (55.8%) followed by the upper extremity (12.6%) which was similar to the prospective study in Lebanon which revealed the lower extremity as the commonest site of occurrence in 89.5% of patients [23].

Effective adjuvant or neoadjuvant regimens of chemotherapy with timely local control with surgery have dramatically improved the prognosis of patients with localized osteosarcoma evidenced by many studies reaching the five-year amore th [27, 28, 30, 31]. The 5-year overall survival with a combination of surgery with chemotherapy for localized primary malignant bone tumors has increased to 60–70% in better setup [6, 29, 32]. The five-year survival rate and event-free survival for localized malignant bone tumors in developed countries even improved to 81% and 68% respectively, whereas the overall survival for metastatic bone tumors was dismal [21–24].

In our study, the 1-year and 5-year overall survival probabilities for all pediatric primary malignant bone tumor patients were 65% 95% CI:0.3–0.56) and 38% (95% CI:0.19–0.47) respectively. The 1-year and 5-year event-free survival probabilities were 55% (95% CI: 0.32–0.73) and 33% (95% CI: 0.10–0.59), which was lower than other studies [33]. The overall survival in our setup was lower than in the other studies; this might be due to the delayed presentation, treatment abandonment, fewer surgical interventions, and limited radiotherapy services.

Previous reviews on primary metastatic osteosarcoma treated with cooperative osteosarcoma study group protocols showed that the overall survival of osteosarcoma was significantly correlated with patient age, site of the primary tumor, number and location of metastases, number of involved organ systems, and histologic response of the primary tumor to preoperative chemotherapy [32, 34, 35]. Our finding revealed that survival status was dependent on the stage of the disease at diagnosis (*p* = 0.023), and the histology of the tumor; either osteosarcoma or Ewing sarcoma (*P* = 0.050. However, the survival status was independent of age, duration of illness, and primary site of occurrence. These findings are supported by similar findings in South Iran [33]. Our study revealed that children who completed the chemotherapy had better survival than those who didn’t complete or started treatment (*P* ≤ 0.015).

In this study, the survival status was unknown for one-fourth of the patients (26.3%); those patients were lost to follow-up, and addressing their status with a phone call was attempted, but the registered phones were not working or picked up during the time of data collection.

### Strengths and limitations of the study

The study was done in one of the biggest tertiary referral hospitals, serving as the only center for pediatric cancer patients till recently, and our study reports patterns and outcomes of primary bone tumors in children. Our study focused on the overall picture of survival status and determinants of outcomes of primary malignant bone tumors rather than delineating and analyzing separately based on the types of primary malignant bone tumors, which might need a large cohort to study the outcomes and determinants of each type of primary malignant bone tumor. The other limitation of this research is that this study didn’t specifically investigate the effects of local control measures on the overall survival rates of children with primary bone tumors, which needs to be addressed in future studies as local control measures such as surgery or radiotherapy may play a significant role in the survival of children with solid cancers.

## Conclusion

In our study, most bone tumor patients seek medical care after an average of five months, and more than one-third of the patients presented with advanced stage (Metastatic disease). The 1-year and 5-year overall survival and event-free survival probabilities for all pediatric primary malignant bone tumor patients were low and emphasis has to be given to improving childhood cancer survival in Low and Middle-Income Countries (LMICs). Future research should identify barriers and psycho-social issues for late presentation, loss to follow-up, and treatment abandonment in children and adolescents diagnosed with primary bone tumors.

## Data Availability

The datasets used and analyzed are available upon reasonable request from the corresponding author.
